# Enhancing bank marketing strategies with ensemble learning: Empirical analysis

**DOI:** 10.1371/journal.pone.0294759

**Published:** 2024-01-11

**Authors:** Xing Tang, Yusi Zhu

**Affiliations:** 1 Institute of Traffic Engineering, Nanjing Vocational University of Industry Technology, Nanjing, Jiangsu, China; 2 School of Mathematics, Sichuan University, Chengdu, Sichuan, China; Chitkara University, INDIA

## Abstract

In order to enhance market share and competitiveness, large banks are increasingly focusing on promoting marketing strategies. However, the traditional bank marketing strategy often leads to the homogenization of customer demand, making it challenging to distinguish among various products. To address this issue, this paper presents a customer demand learning model based on financial datasets and optimizes the distribution model of bank big data channels through induction to rectify the imbalance in bank customer transaction data. By comparing the prediction models of random forest model and support vector machine (SVM), this paper analyzes the ability of the prediction model based on ensemble learning to significantly enhance the market segmentation of e-commerce banks. The empirical results reveal that the accuracy of random forest model reaches 92%, while the accuracy of SVM model reaches 87%. This indicates that the ensemble learning model has higher accuracy and forecasting ability than the single model. It enables the bank marketing system to implement targeted marketing, effectively maintain the relationship between customers and banks, and significantly improve the success probability of product marketing. Meanwhile, the marketing model based on ensemble learning has achieved a sales growth rate of 20% and improved customer satisfaction by 30%. This demonstrates that the implementation of the ensemble learning model has also significantly elevated the overall marketing level of bank e-commerce services. Therefore, this paper offers valuable academic guidance for bank marketing decision-making and holds important academic and practical significance in predicting bank customer demand and optimizing product marketing strategy.

## 1. Introduction

With the rapid advancement of internet and information technology, banks are increasingly diversifying their marketing strategies and expanding their efforts across both offline and online channels by utilizing various new media platforms [1–3]. Marketing ability is not only related to the bank’s customer acquisition and retention, but also related to its brand influence, market share and profitability. But how to effectively predict and improve their own marketing ability? At present, there are few comprehensive models or tools that can comprehensively predict the marketing ability of banks. The existing research focuses more on a single factor or local problem, lacking a global perspective. Therefore, it provides an efficient and data-driven forecasting tool for the banking industry to improve the accuracy and efficiency of marketing decisions. This is of great significance for the banking industry to succeed in the highly competitive market [4, 5]. As China’s economy continues to grow, the number of commercial institutions in the country is on the rise. Consequently, commercial banks find themselves in increasingly fierce competition. Furthermore, the rapid expansion of online financial services is leading to a significant decline in reliance on offline banking customers, which in turn is giving rise to a growing number of customer-related challenges. One of the primary tasks facing commercial banks today is the need to maintain positive relationships with their existing offline clients while also enhancing their marketing capabilities to meet evolving customer demands. To better manage these customer relationships and navigate the challenges posed by the rapid development of the internet, data mining technology has been widely adopted across various aspects of life [6]. In marketing, banks need to accurately predict customers’ needs, behaviors and reactions to formulate relevant strategies and plans. Ensemble learning can significantly improve the prediction accuracy by combining the prediction results of several basic models [7]. This is very important for banks, because accurate forecasting can help them better locate customers and launch targeted products and services, thus improving sales efficiency and customer satisfaction. This provides strong support for the creation of a bank marketing capability model.

This paper introduces a customer demand learning model based on a financial dataset, which advances the overall effectiveness of bank e-commerce by simulating and predicting actual customer business needs. It is grounded in research on internet technology, ensemble learning models, and an analysis of traditional banking marketing models. The following outlines the paper’s primary logical structure: Section 1 provides an overview of the historical development of ensemble learning and internet technologies. Section 2 consolidates recent research on ensemble learning models and the growth of bank marketing capabilities. In Section 3, a forecasting system for a bank’s marketing capabilities is established through data analysis of bank customers. The model’s predictions and experimental findings are discussed in Section 4. Finally, Section 5 presents the research conclusions after a thorough examination of the data results. The paper is a useful source of information for encouraging the commercial banks’ marketing skills to grow intelligently.

## 2. Recent related work

### 2.1 Recent work on ensemble learning model

Mao et al. (2019) [8] investigated the maximization and diversity of ensemble learning through transformation. They developed a weighted ensemble learning technique that simultaneously maximized variety and individual accuracy, contributing to the latest advancements in ensemble learning models. In the proposed framework, several basic learners are combined and then turned into linear transformations of each of these basic learners. The optimal weight is then determined by pursuing the best projection direction of the linear transformations. The performance of the suggested method is demonstrably improved by comparison to other ways according to experimental findings. Dong et al. (2020) [9] analyzed ensemble learning using cutting-edge computer science technology and identified ensemble learning as a research hotspot. The findings demonstrated that the suggested model can successfully construct an effective knowledge discovery and mining system by integrating data fusion, data modeling, and data mining into a single framework. Alam et al. (2020) [10] proposed a novel dynamic ensemble learning algorithm based on neural network fusion after conducting a study on dynamic ensemble learning algorithms for neural networks. The experimental findings supported the notion that the dynamic neural network ensemble produced by the new dynamic ensemble learning has a suitable design, a diverse population, and a high degree of generalizability. Chen et al. (2021) [11] employed deep learning technology to investigate network security in smart cities. The research demonstrated that the proper design based on deep learning method was very important to protect smart city networks through the analysis and comparison of deep learning models such as Boltzmann machine, restricted Boltzmann machine, Deep Belief Network (DBN), Recurrent Neural Network (RNN), and convolutional neural network (CNN). Matloob et al. (2021) [12] contributed to the field by providing concise insights into the latest trends and developments in ensemble learning for software defect prediction. They also predicted and studied software defects based on ensemble learning. The performance of the model’s predictions was evaluated using the ensemble learning technique. The study demonstrated that feature selection and data sample were essential preprocessing stages that could enhance the effectiveness of an ensemble classifier. By system mapping study and cross-benchmark evaluation, Tama et al. (2021) [13] examined the ensemble learning technology of intrusion detection system and made a thorough empirical evaluation on the most recent advancements in ensemble learning technology. The findings demonstrated that by ensemble learning from intrusion detection systems, the model’s prediction accuracy was greatly increased.

Furthermore, in regards to the framework system of domain adaptive ensemble learning, Zhou et al. (2021) [14] studied the unifying framework of domain adaptive ensemble learning, and studied the non-expert ensemble learning from other sources to provide supervision signals through the analysis of multi-source unsupervised domain adaptation. Domain adaptable ensemble learning has significantly improved on these two concerns, and typically offers several benefits according to a huge number of trials. In their study of the forest fire detection system based on ensemble learning, Xu et al. (2021) [15] integrated two different learners, Yolov5 and EfficientDet, to complete the fire detection process. Tests on data sets demonstrated that the suggested strategy reduced the false alarm rate by 51.3% without adding any extra time and increased detection performance by 2.5% to 10.9%. Lin et al. (2021) [16] investigated the plug-in hybrid vehicle’s energy management method using ensemble learning speed prediction. According to the verification results, the suggested technique outperformed the benchmark strategy in terms of great fuel efficiency over a variety of driving cycles. Zhang et al. (2022) [17] researched and developed a method based on ensemble learning, studied the slope stability prediction technology based on that technology, predicted the slope stability by introducing random forest and extreme gradient lifting, and then applied that method to the slope stability prediction and research in Chongqing. The findings suggested that the ensemble learning-based proposed method offered a potential way to appropriately capture the slope state. Abbasi et al. (2022) [18] used ensemble learning to determine and study identity. Their experimental findings revealed that the ensemble learning approach suggested in the study improved accuracy by 14.2%. Yin et al. (2022) [19] conducted research on the ensemble learning model of the Bayesian optimization technique. By combining the extreme gradient lifting and random forest models in an ensemble learning approach, the XGBoost model outperformed the random forest model in terms of accuracy, precision, recall, F1 score, and kappa coefficient. The study could serve as a guide for creating maps of mineral prospects and perfecting ensemble learning models. Nguyen et al. (2022) [20] devised a computer technique to virtually screen possible anti-cancer drugs and used ensemble learning paired with evolutionary computation to identify anti-cancer natural products. They also built an ensemble computing framework through machine learning. According to the study, using the ensemble model to identify natural compounds with anticancer activity has real-world applications.

The rapid development of the Internet of Things (IoT) and information technology (IT) has highlighted the inadequacy of single data classifiers in handling the demands of big data processing. Ensemble learning technologies address this challenge by employing multiple data classifiers, enabling stacking, additional processing, and prediction generation from model data. This approach not only enhances prediction accuracy but also increases the effectiveness and scalability of data processing.

### 2.2 Progress and related work on bank marketing ability

The potential sources of climate funding in underdeveloped nations were compared by Banga (2019) [21] with regard to the assessment and analysis of banks’ marketing capabilities. The findings of the study on banks’ marketing capabilities and green bonds demonstrated that, thanks to investors’ growing climate consciousness, green bonds were on the increase in both developed and emerging markets. However, in developing nations, the green bond market was still in its infancy, and its full potential had not been fully realized. Boating (2019) [22] investigated the link between online relationship marketing and customer loyalty, collected experimental data by looking into 429 Ghanaian retail bank clients, and used structural equation modeling technology to assess the findings. The study demonstrated that in addition to the online technologies employed, banks’ online relationship activities must also transmit pertinent and beneficial signals to support the promotion of their marketing skills. In order to gather information, Nazaritehrani et al. (2020) [23] researched the growth and market share of e-banking channels in developing nations and created a questionnaire. The expert judgments and the Kronbach technique were used to evaluate the scale’s validity and reliability. The study noted that although there was a statistically significant association between a bank’s market share and the growth of its marketing capabilities, the outcome was not significant. Asnawi et al. (2020) [24] investigated the significance of customer satisfaction and loyalty in Indonesia and its impact on Islamic banks, testing the theory by compiling information from 280 Indonesian bank clients. Managers can specifically tailor their services for Muslim customers by evaluating the Islamic banks’ customer service standards. The gap between what customers expect from the services offered and what they actually receive can be reduced and customer satisfaction can also be increased while establishing the bank’s marketing strategy. Mulia et al. (2021) [25] investigated the effect of customer intimacy in enhancing customer loyalty of Islamic banks, and the information was gathered through self-management investigation techniques. Meanwhile, data analysis employed multivariate linear regression and multivariate analysis of variance. The findings indicated that loyalty was impacted both directly and indirectly by customer closeness.

Several factors influence a bank’s effectiveness in marketing itself within the financial and commercial markets. Arjun et al. (2021) [26] conducted research into the evolving landscape of bank intelligence in emerging countries, focusing on the distinct intelligent decision support model of the banking industry. Their study examined critical elements that impact a bank’s marketing capability and demonstrated how the advancement of artificial intelligence (AI) and Internet of Things (IoT) technologies could enhance these capabilities. Rahmayati (2021) [27] empirically analyzed the competitive strategy of Islamic banking, gathering data from written sources. The study’s outcomes revealed that banks can increase customer satisfaction through the implementation of proactive marketing methods. Boustani (2022) [28] investigated the impact of AI on customers and staff in banks across developing Asian nations. Using a model based on quantitative research and a fictitious regression model for analysis, the study found that AI elevated the quality of bank transactions, serving as a practical benchmark for predicting and assessing a bank’s marketing potential. In order to get experimental data, Mogaji et al. (2022) [29] conducted semi-structured interviews with 47 bank managers from both developed and developing nations. They researched the AI technology connected to marketing financial services. The conceptual framework of AI connected to financial service marketing presented in this paper captured and emphasized the interaction between clients, banks, and outside stakeholders. The research was a useful source of information for enhancing bank marketing capabilities. With regard to Indian banking, Sharma et al. (2022) [30] conducted qualitative and quantitative research, proposed a conceptual model for the green banking effort, and examined the impact of the green banking initiative on bank marketing prowess. The study demonstrated that the green bank strategy contributed favorably to improving the green image and regaining client trust. Galletta et al. (2022) [31] used samples from 205 Nordic listed businesses during the period of 2002 to 2020 to investigate gender diversity and sustainable performance in the banking sector, as well as the relationship between CSR and environmental management. The research showed that the correlation between gender diversity of board of directors and sustainable performance is more obvious in the carbon-intensive industry sub-sample.

In conclusion, the era of big data has ushered in changes in the marketing model, gradually replacing the traditional telemarketing model with the internet marketing model due to the substantial increase in data volume. To better understand the evolution of commercial banks’ marketing capabilities and identify the algorithm models best suited for accurate marketing of bank wealth management products, further analysis of the user structure of these products is necessary. This analysis is crucial for advancing business operational methods.

## 3. Prediction and evaluation model of bank marketing big data distribution based on ensemble learning

### 3.1 Ensemble learning model and bank marketing

**Fig 1:**
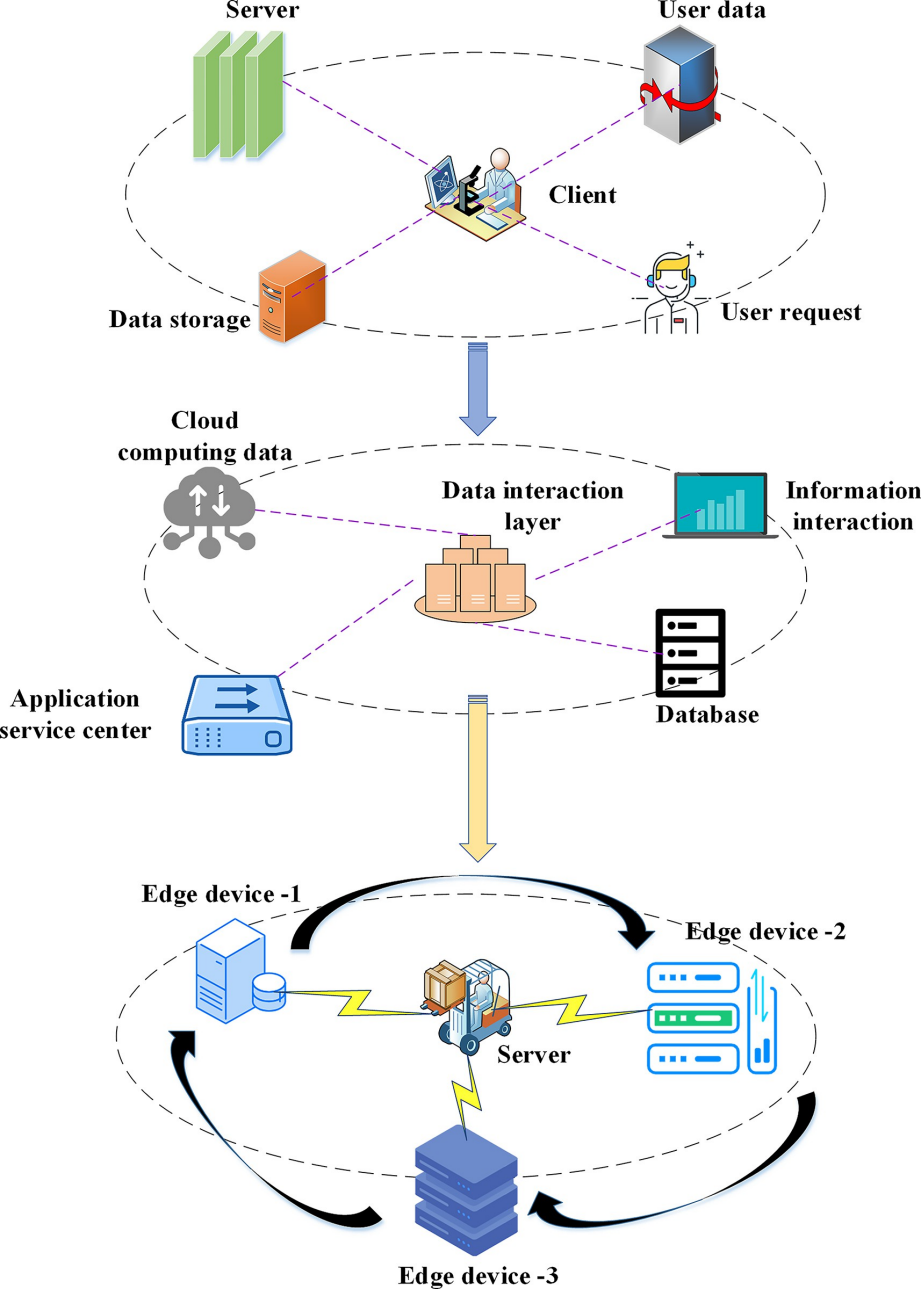
Data interaction structure diagram of ensemble learning model.

As IoT technology has advanced, the precision of machine learning tasks has become more crucial than their processing speed [32]. By developing a robust learning model structure within the ensemble learning framework, machine learning tasks can be effectively completed. In assessing participants’ marketing skills based on data, the expected value of each model evaluator generates a random combination, typically resulting in more precise predictions than those of individual participants. A hierarchical control decision-making structure model for learning monitoring is created to handle classification, regression, and preprocessing tasks in advanced ensemble learning concepts. When the loss function is straightforward, the data optimization phase can be relatively straightforward, involving the error quadrant function or exponential function for data processing. The ensemble learning model’s data interaction mechanism among the user layer, server layer, and data interaction layer is examined. Fig 1 illustrates the system architecture of the ensemble learning model.

In Fig 1, in the context of bank marketing ability prediction, customers can be the marketing team, analysts, business decision makers or other relevant personnel of the bank. Customers can connect with the data interaction layer through applications, user interfaces or other means, and request forecasts or other relevant information from the server. The data interaction layer is a key component to connect the client and the server. It plays a bridge role in data transmission, communication and interaction. A server is a computer system or cloud service that stores and hosts an ensemble learning model. It is responsible for receiving requests from customers, and then calling or running corresponding models to generate prediction results.

### 3.2 Bank customer demand and financial big data collection and analysis

In the banking industry, accurately predicting whether customers will make time deposits is of utmost importance when analyzing real customer data through the banking marketing system [33]. Big data collection is an Extract-Transform-Load (ETL) operation on data sources. When collecting financial data, due to the complexity of data sources, before data analysis, it is necessary to extract the data needed for precise marketing from the complex original data format through extraction technology.

The financial data extraction in this paper is a process of extracting the required data from the big data of existing marketing systems in banks (China Merchants Bank, China Construction Bank, China Agricultural Bank and China Industrial and Commercial Bank) through ETL tools. Data extraction is summarized according to the agreed XML format and Service format. First, it is filtered and analyzed, and then the data is transformed into information and knowledge. The methods of data extraction include total extraction and incremental extraction. Total extraction is equivalent to data migration or copying, which extracts the data from the table or view in the data source from the database and converts it into the format recognized by ETL tools. Incremental extraction refers to extracting new or modified data from the database. Incremental extraction is more widely used in ETL tools than full extraction of data, and how to capture changing data is the key to incremental extraction of data. Firstly, the total data of 2020 and 2021 are crawled from different subsystems and stored in the data warehouse. Meanwhile, ETL tools extract customer data, sales data and regional data needed for precise marketing from the big data of the existing marketing system of banks. Then, in the end, this paper extracts a total of 25317 bank sales data through the above extraction methods. When collecting and using financial data, it strictly abides by ethical and privacy standards, respect the confidentiality and sensitivity of financial data, and ensures the legal acquisition and use of data.

**Fig 2:**
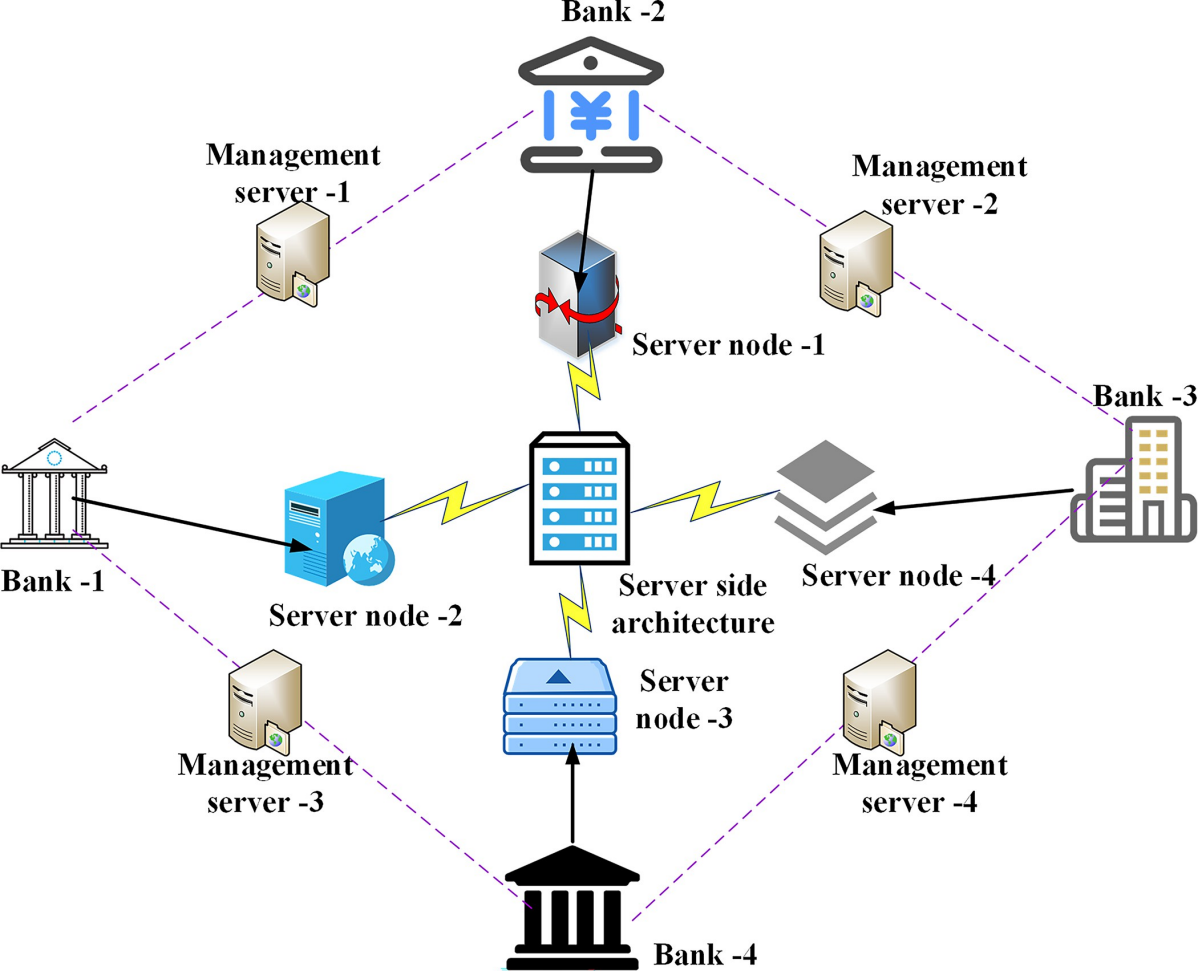
Structure of customer data analysis and management system.

The process of model interpretable analysis begins with data analysis. Firstly, the research requires a comprehensive understanding of the data, encompassing an assessment of the overall data distribution and an analysis of feature relationships. The second step involves the use of data visualization techniques to meticulously characterize and analyze the data before constructing a data-driven self-service framework. By considering both the correlations within the dataset and those between data elements and target variables, uncovering hidden patterns within financial datasets can significantly enhance the model’s predictive capacity. This optimization of information data for the features required by machine learning models holds substantial reference value. Fig 2 illustrates the structure of the customer data analysis and management system, providing an overview of the server-side structure and management framework for bank marketing businesses.

In Fig 2, in this structure diagram, the client is connected to the bank and the management server through the network, and can perform various data analysis and management tasks. The bank is responsible for data analysis and model training, while the management server is used for system configuration and monitoring. Server nodes provide computing and storage resources to support the operation of the system. Finally, the management server provides centralized management and control of the system to ensure the stability and maintainability of the system.

### 3.3 Analysis on the construction of bank marketing ability forecasting model based on ensemble learning

Ensemble Learning, as one of the machine learning algorithms, completes the learning task by combining multiple individual learners. These learners have the same type and different types. The same type is called homogeneous and the different types are called heterogeneous [34]. Usually, homogeneous algorithms usually include Boosting algorithm and Bagging algorithm, while heterogeneous algorithms combine different types of classifiers by adopting some fusion strategy, such as Stacking algorithm. Although Stacking algorithm combines the advantages of multiple models, the calculation cost of Stacking is high, and cross-validation and the construction of multiple base models make it consume a lot of calculation resources and time. Therefore, this paper mainly analyzes lifting method and bagging method.

The advantage of Boosting algorithm is that it can combine different learners with weights, thus improving the performance of the model. At present, the widely used Boosting algorithms include GBDT algorithm, XGBoost algorithm and LightGBM algorithm. Among them, Extreme Gradient Boosting (XG Boost) is a machine learning system. It adopts distributed gradient lifting algorithm, which can effectively improve the accuracy and efficiency of classification, regression and feature selection. The objective function is shown in Eq (1):

$$obj^{\left(t\right)}=\sum_{i=1}^{n}L(y_{i},{\hat{y}}_{i}^{\left(t\right)})+\sum_{i=1}^{t}\Omega (f_{i})$$ (1)

*L* refers to the loss function and Ω refers to the positive term. For a tree containing (*t*-1) integration trees, the (*t*)th feature decision tree can be expressed as Eq (2):

$${\hat{y}}_{l}^{\left(t\right)}={\hat{y}}_{l}^{\left(t-1\right)}+f_{t}(x_{i})$$ (2)

${\hat{y}}_{l}^{\left(t-1\right)}$ refers to the predicted value given by the model in step (t-1), it is a known constant. *f*_*i*_(*x*_*i*_) refers to the predicted value of the feature tree to be added this time. In this case, the objective function is shown in Eq (3).


$$obj=\sum_{i=1}^{n}L(y_{i},{\hat{y}}_{l}^{\left(t\right)}+f_{t}(x_{i}))+\Omega (f_{t})+constant$$ (3)

According to Taylor formula, the function *f*(*x*+Δ*x*) is expanded in Taylor’s second order at point *x*, and the Eq (4) can be obtained:

$$f(x+\Delta x)\approx f(x)+f'(x)\Delta x+\frac{1}{2}f''(x)\Delta x^{2}$$ (4)

Taylor’ formula is brought into the objective function of XGBoost, where *x* corresponds to the predicted value ${\hat{y}}_{l}^{\left(t-1\right)}$ of the first (*t*-1) feature tree and Δ*x* corresponds to the *t* tree *f*_*i*_(*x*_*i*_) being trained, and the objective function is rewritten as Eq (5):

$$obj^{\left(t\right)}=\sum_{i=1}^{n}\left[L(y_{i},{\hat{y}}_{l}^{\left(t-1\right)})+g_{i}f_{t}(x_{i})+\frac{1}{2}h_{i}f_{t}^{2}(x_{i})\right]+\Omega (f_{t})+constant$$ (5)

The objective function obtained by removing the constant term is shown in Eq (6).


$$obj^{\left(t\right)}=\sum_{i=1}^{n}\left[L(y_{i},{\hat{y}}_{l}^{\left(t-1\right)})+g_{i}f_{t}(x_{i})+\frac{1}{2}h_{i}f_{t}^{2}(x_{i})\right]+\Omega (f_{t})$$ (6)

Compared with Boosting algorithm, Bagging algorithm aims at reducing the variance of the model, that is, reducing the sensitivity of the model to data, thus improving the generalization ability of the model. Random forest introduces more randomness on the basis of Bagging algorithm. By randomly selecting some features every time a node is split, the correlation of the model is reduced and the diversity of the model is improved. Therefore, random forest is more suitable for data sets that are unstable or susceptible to noise. Therefore, random forest is more suitable for data sets that are unstable or susceptible to noise.

Suppose a random forest model contains T decision trees, and the test sample *x* needs to be classified into category *y*. Assuming that each decision tree is independent of each other, the whole random forest can be regarded as the average of multiple decision tree classifiers, as shown in Eq (7):

$$f(x)\frac{1}{T}\sum_{i=1}^{T}f_{i}(x)$$ (7)

*f*_*i*_(*x*) refers to the classification result of sample *x* by the ith decision tree. Let the variance of each decision tree be $\sigma_{i}^{2}$, then the variance of the whole random forest is shown in Eq (8):

$$\sigma_{i}^{2}=\frac{1}{T^{2}}\sum_{i=1}^{T}\sum_{j=1}^{T}Cov(f_{i}(x),f_{j}(x))$$ (8)

$Cov(f_{i}(x),f_{j}(x))$ can be expressed as Eq (9):

$$Cov(f_{i}(x),f_{j}(x))=E[\left(f_{i}(x),f_{j}(x)\right)]-E[f_{i}(x)]E[f_{j}(x)]$$ (9)

Because the classification prediction results of each decision tree are independent of each other, there exists Eq (10).


$$E[\left(f_{i}(x),f_{j}(x)\right)]=E[f_{i}(x)]E[f_{j}(x)]$$ (10)

When *i* and *j* take different values, $E[\left(f_{i}(x),f_{j}(x)\right)]$ is shown in Eq (11):

$$E\left[(f_{i}(x),f_{j}(x))\right]=E\left[f_{i}(x)\right]E\left[f_{j}(x)\right]=\{\begin{array}{cc} E{\left[f(x)\right]}^{2} & i\neq j\\ E{\left[f_{i}(x)\right]}^{2} & i=j \end{array}$$ (11)

Therefore, the variance of random forest can be rewritten as Eq (12):

$$\sigma_{}^{2}=\frac{1}{T}\sigma_{i}^{2}+\frac{1}{T^{2}}\sum_{i\neq j}Cov(f_{i}(x),f_{j}(x))$$ (12)

Among them, the first term is the variance of a single decision tree, and the second term is the covariance between decision trees. Because of the differences between decision trees, the covariance is often small and can usually be ignored. The above equation can be approximated as Eq (13):

$$\sigma_{}^{2}=\frac{1}{T}\sigma_{i}^{2}$$ (13)

The variance of random forest is approximately equal to the variance of a single decision tree divided by the number of decision trees. It shows that random forest can greatly reduce the variance of model prediction results and obtain better classification accuracy.

**Fig 3:**
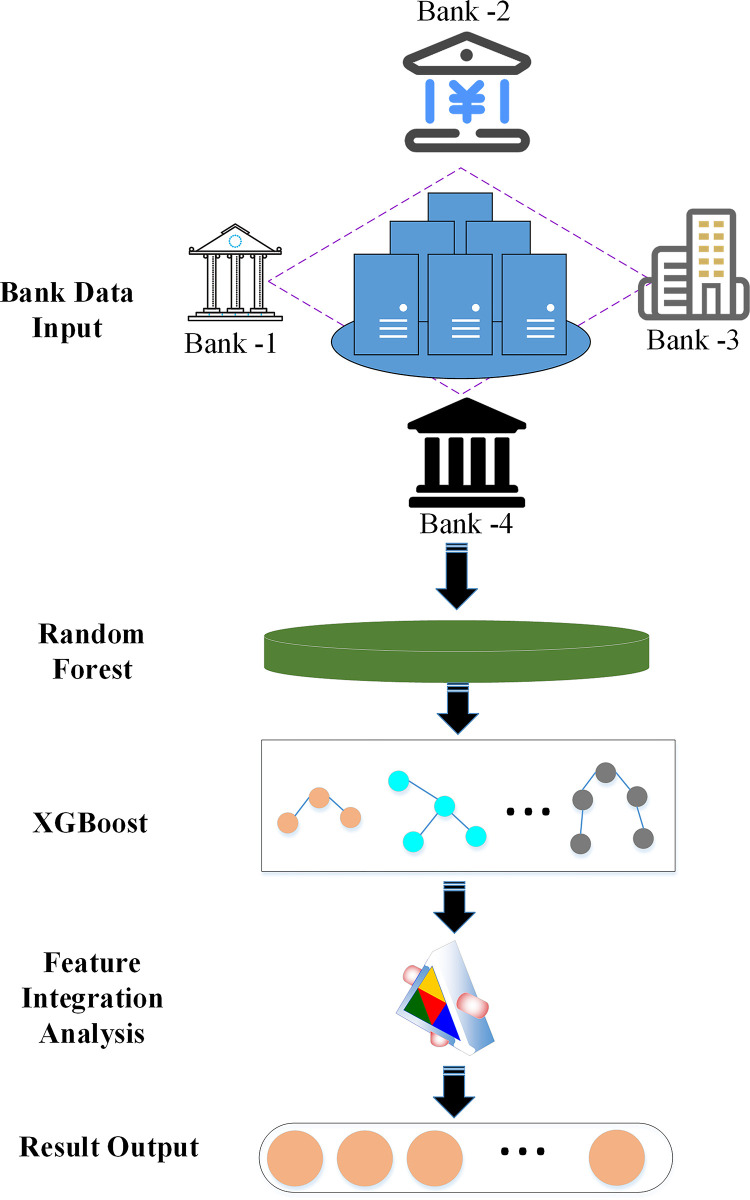
Schematic diagram of bank marketing ability prediction model framework based on ensemble learning.

In order to improve the algorithm, this paper combines XGBoost algorithm in ensemble learning with random forest algorithm and applies it to the prediction of bank marketing ability. Fig 3 describes the forecasting model of bank marketing ability based on ensemble learning.

In Fig 3, the model firstly uses the random forest algorithm to make feature selection and preliminary prediction for the bank marketing system, and then takes the output of the random forest as the input feature of XGBoost, and uses XGBoost to train a new model to better capture the complex relationships and patterns in the data. Finally, the forecast results of bank marketing ability are integrated and output.

**Fig 4:**
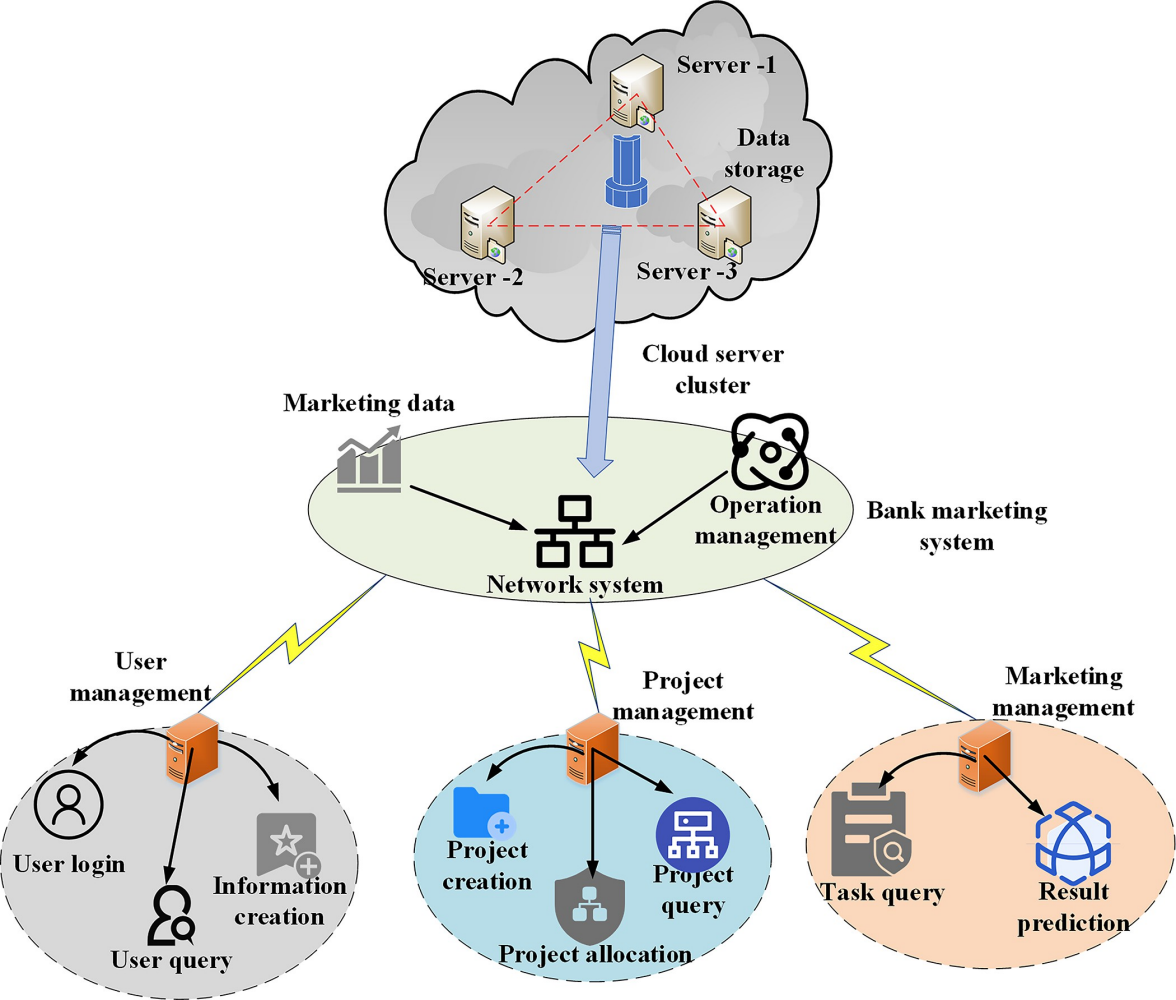
Network system structure of bank marketing ability management.

In this model, accurate analysis of bank marketing opportunities typically uses superposition, under sampling, and thorough sampling methods. By increasing the number of samples, overlapping sampling corrects for positive sampling, but this necessarily results in a huge amount of noise data [35]. By reducing the proportion of most samples, the primary tenet of an under-sampling technique is to enhance the classification effect of the model in a few categories. Regrettably, this approach may slightly reduce the model’s accuracy prediction rate while also reducing the sample size. These two sampling techniques are combined to create a comprehensive sampling strategy. Overlapping sampling involves adding a small number of samples to the training set, while under sampling removes more incorrect sample data. Extensive grid search for multiple parameters could potentially impact the model’s performance. The random forest algorithm must be utilized to look up machine learning parameters in order to resolve this issue. It is required to input the weight of participants in the model to increase the marketing forecasting capability of banks in accordance with the accuracy of the prediction results of the basic model because the parameter accuracy of each ensemble learning model differs. User management, project management, and sales management make up the bulk of the network system for managing bank marketing capability. Fig 4 depicts the precise structural framework.

In Fig 4, the core part of the network architecture includes user management, project management and sales management, which work together to help banks better organize and coordinate marketing activities, improve marketing capabilities, meet customer needs and achieve sustainable growth.

### 3.4 Experimental evaluation

**Fig 5:**
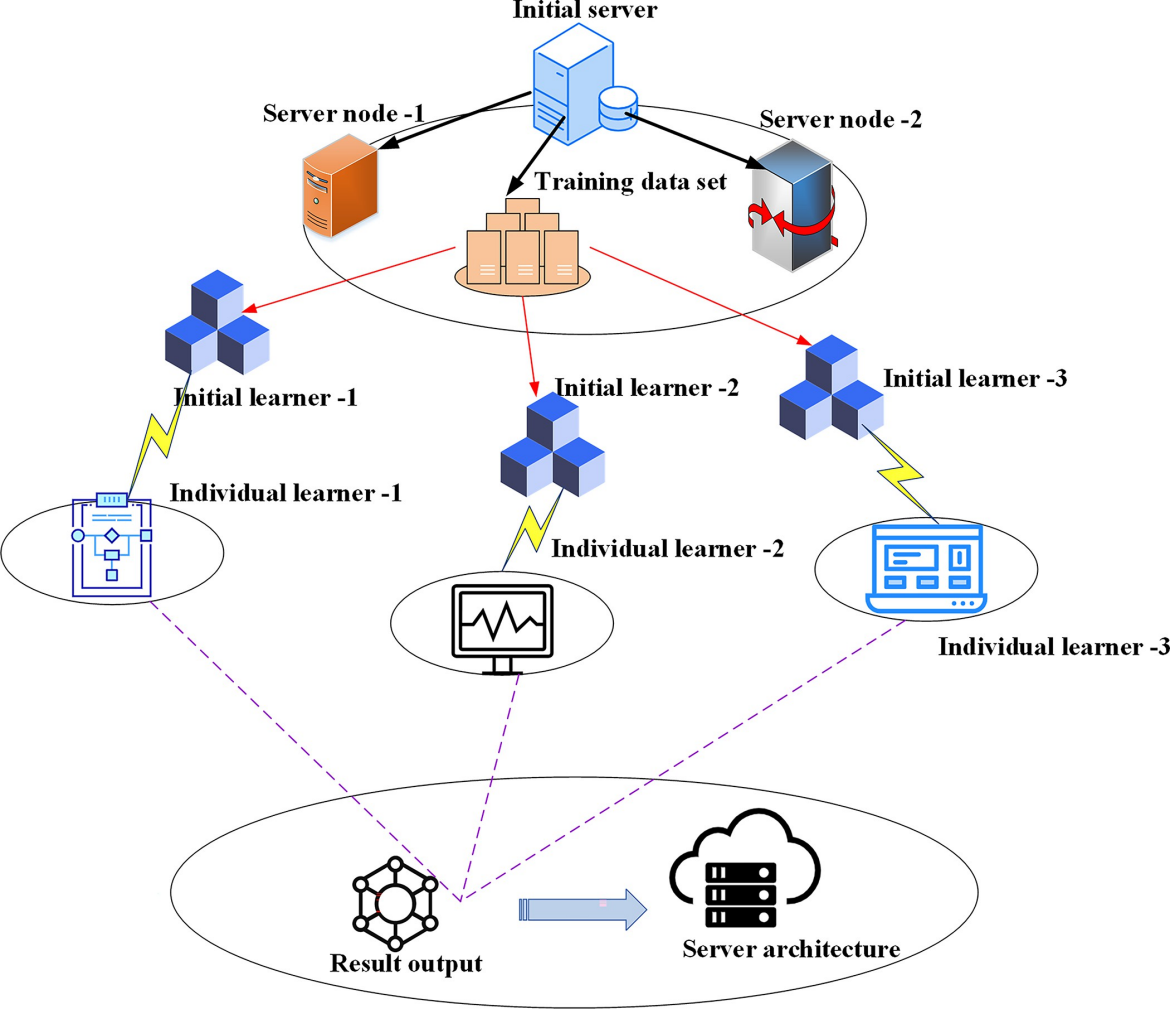
Data processing and operation flow of initial learner.

When analyzing the performance of the bank marketing ability prediction model based on ensemble learning, the input data of the model must be cleaned and preprocessed first to obtain appropriate prediction results. The data processing process of the server and the model is analyzed, and the data processing process of the established initial learner is shown in Fig 5.

In Fig 5, the model’s input data must first be cleaned and preprocessed for the model to obtain appropriate prediction results. The first steps involve filling in the gaps in the data, cleaning out the noise, and addressing the imbalance between the positive and negative sample data. Second, in order to create a new combination of structural features, the original features of the data must be processed and synthesized. Finally, using a suitable algorithm model, feature extraction and model construction are carried out.

When evaluating the performance of the model, a comparative experiment is adopted. In this process, the data set is divided into training set and test set according to the ratio of 7:3, and the basic learner is cross-verified by 50%. In the model, the super parameter optimization of XGBoost algorithm is as follows: max_ depth is 4, gamma is 0.7, min_ child- weight is 5, Reg_ alpha is 0.01, reg_lambda is 100, and num_leaves is 15.

In contrast experiments, random forest, as an ensemble learning method, can reduce the variance of the model and improve the generalization performance by constructing multiple decision trees and combining their prediction results. Decision tree, Support Vector Machine (SVM) and K-nearest neighbor (KNN) are one of the machine learning algorithms respectively, and ensemble learning also belongs to the machine learning algorithm. Therefore, the model algorithm proposed in this paper is compared with the model algorithm proposed by decision tree, random forest, SVM, KNN and Yin et al. (2022), and evaluated from the indicators of accuracy, precision, recall rate, F1 value, data transmission delay and overall system delay. Among them, the accuracy of all experiments is the accuracy of 10 cross-validation under the optimal parameters, and the dispersion degree of accuracy is reflected by calculating the standard deviation of data.

The model’s performance evaluation findings demonstrate the superiority of the ensemble learning-based marketing ability prediction model. The bank marketing system may conduct targeted marketing for consumers and strengthen the relationship between customers and banks by contrasting the prediction models of random forest and SVM as well as contrasting the model suggested in this paper with these models.

## 4. Results and discussion

### 4.1 Prediction performance of different types of bank marketing ability prediction models

**Fig 6:**
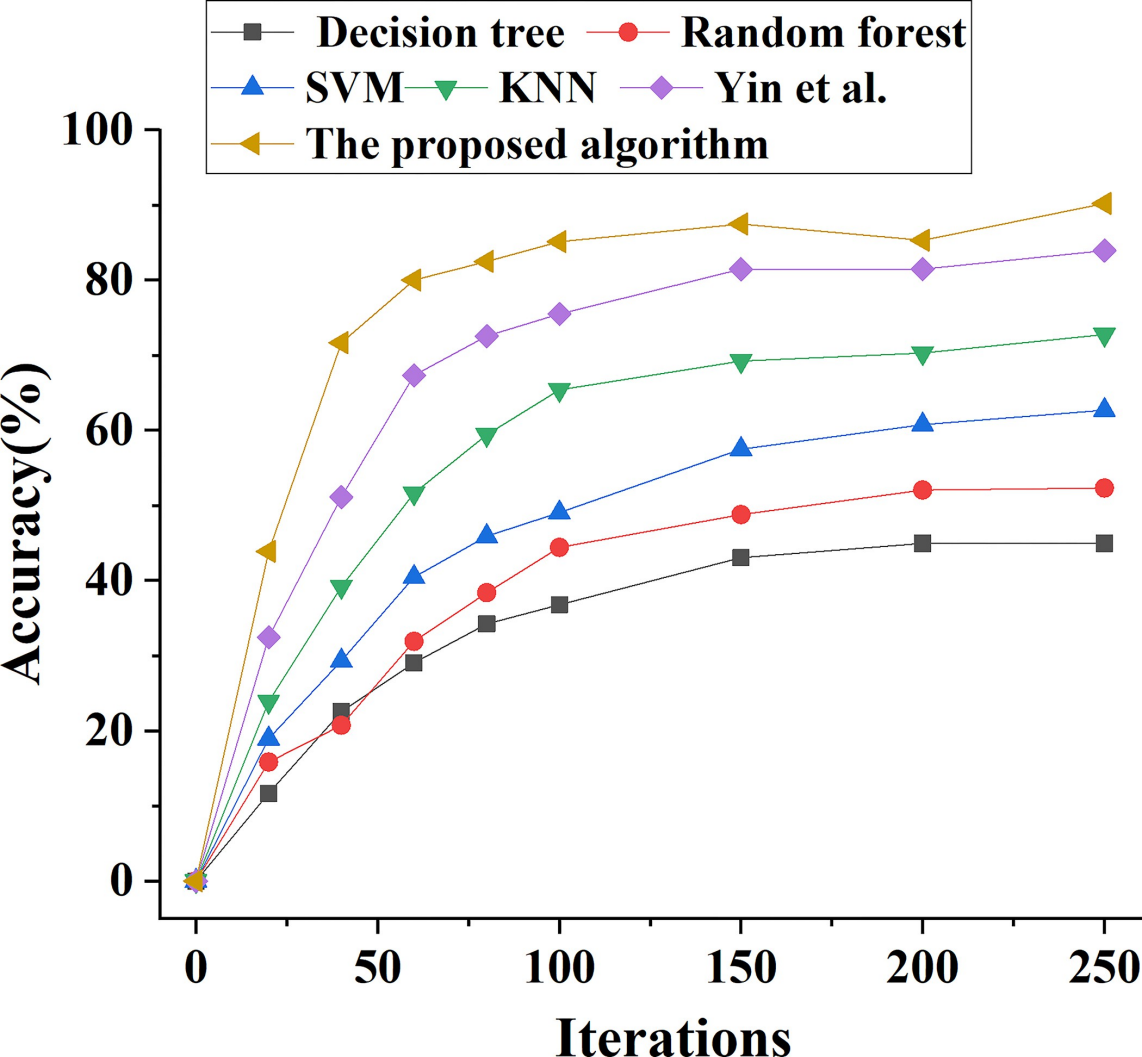
Different types of models predict the accuracy value change curve of bank marketing ability.

**Fig 7:**
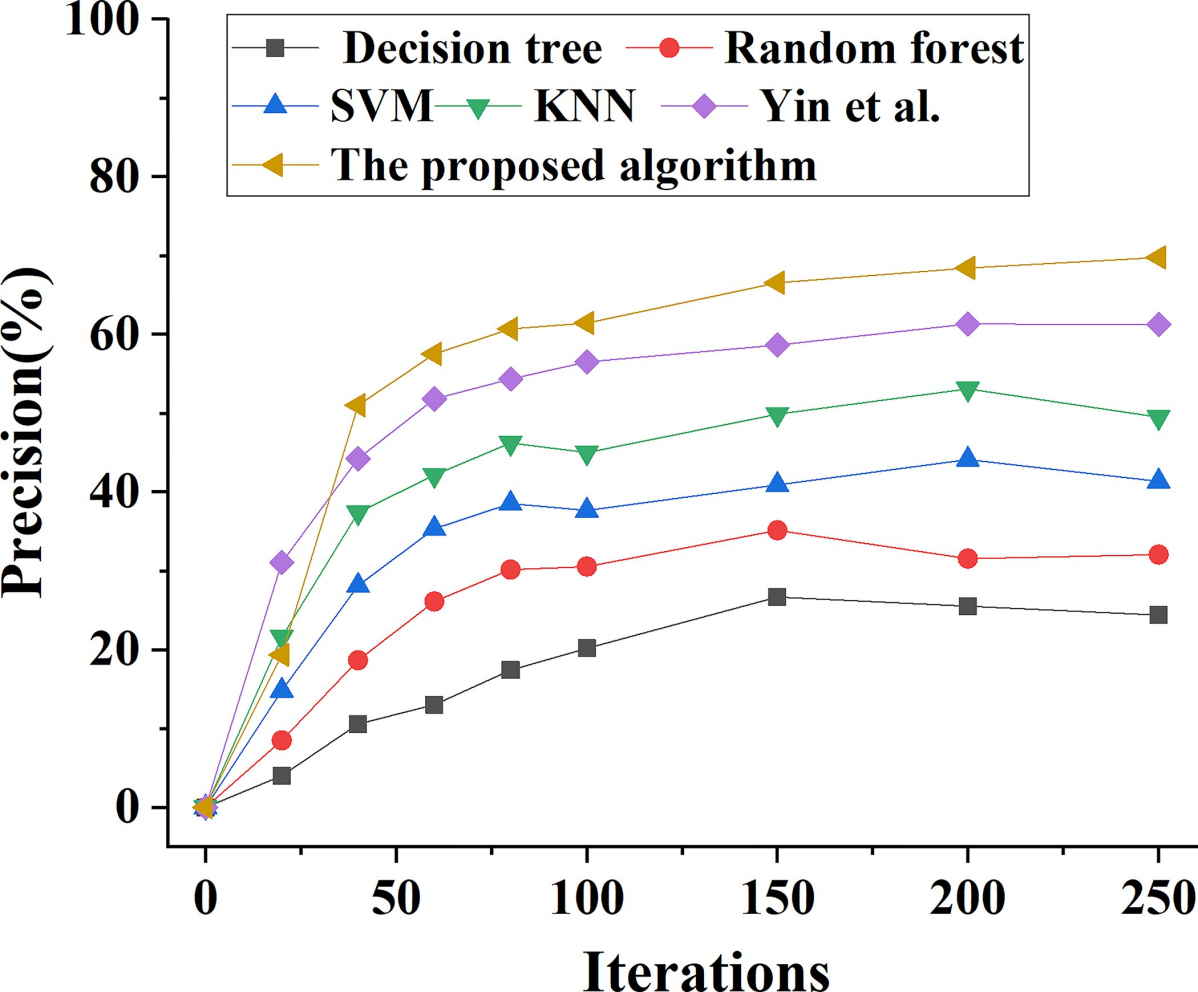
Different types of models predict the precision change curve of bank marketing ability.

**Fig 8:**
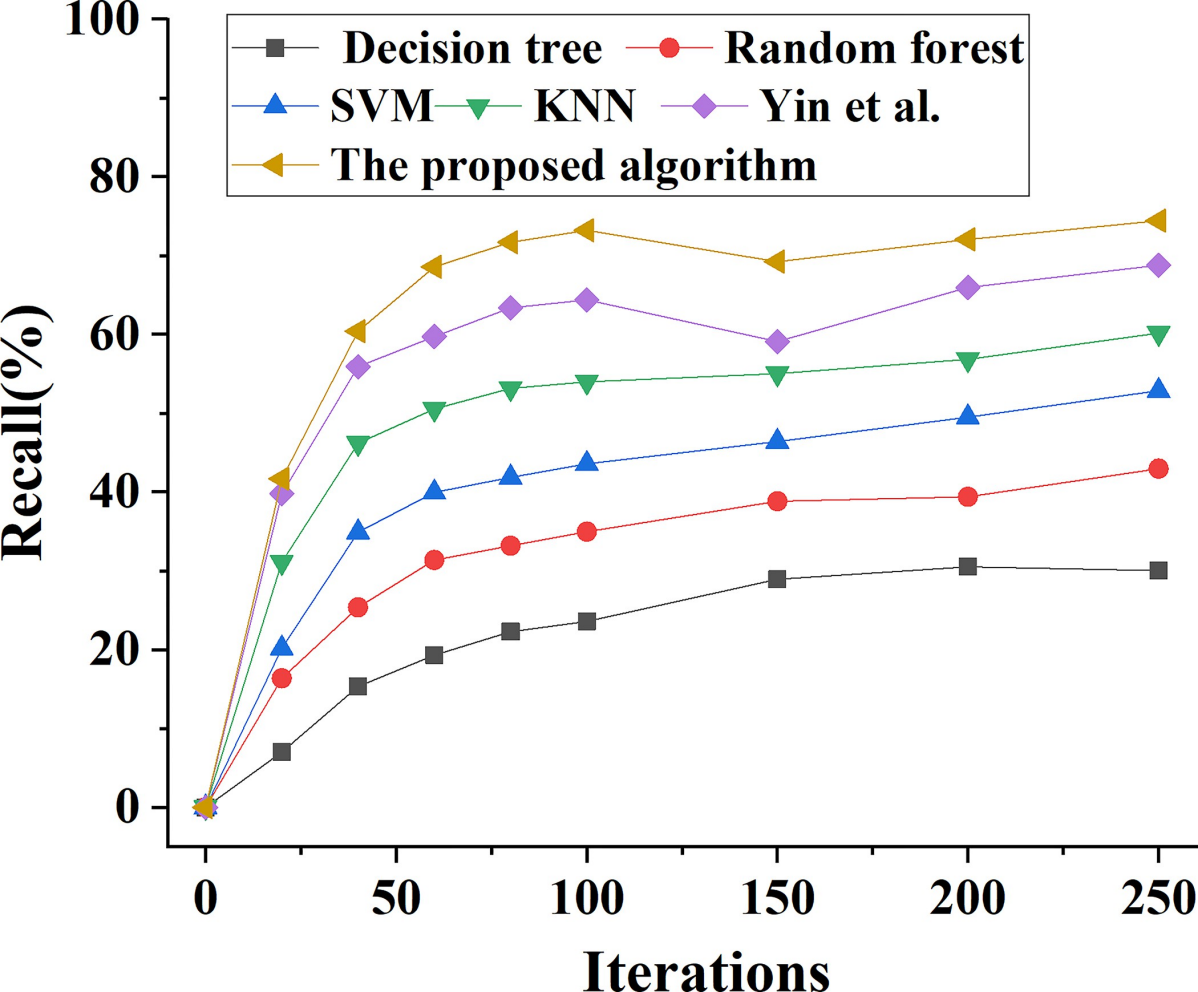
Different types of models predict the change curve of recall rate of bank marketing ability.

**Fig 9:**
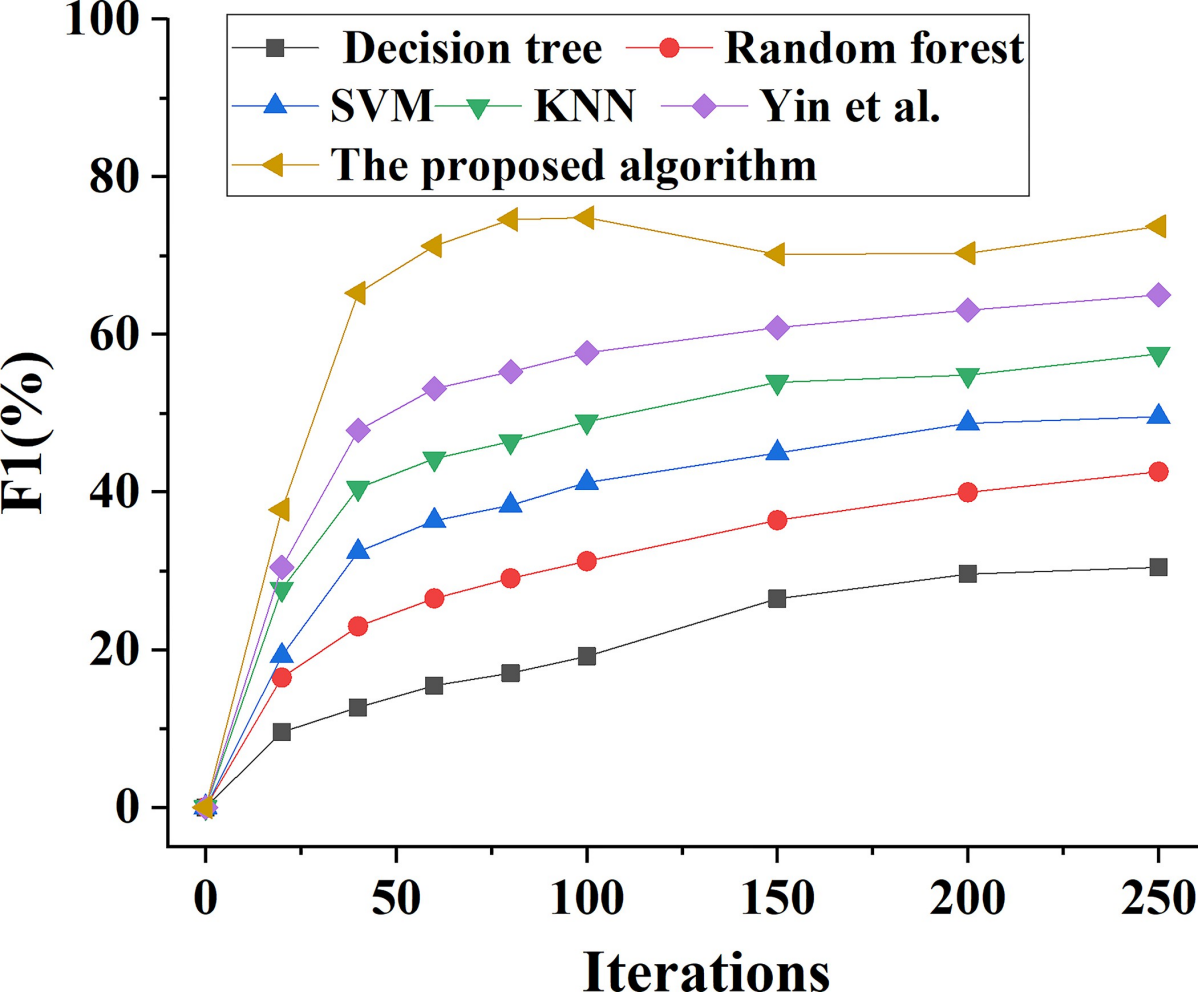
Variation curve of F1 value predicted by different types of models for bank marketing ability.

The performance of the ensemble learning model proposed in this paper is compared with the performance of Decision tree, Random Forest, SVM, KNN and the model algorithm proposed by Yin et al. (2022). The change trend of model prediction accuracy performance is shown in Fig 6. This comparison is done to assess the overall prediction performance of various types of bank marketing ability prediction models. In addition, Figs 7–9 demonstrate the analysis model’s prediction precision rate, recall rate, and F1 value trend, respectively.

Fig 6 shows that with the increase of model iterations, the prediction accuracy of various bank marketing ability prediction models first rises rapidly, and then tends to be stable. When the model is iterated for 100 times, the accuracy of each model tends to be stable, in which the prediction accuracy of decision tree model is 37%, that of SVM model is 49%, and that of suggested model is 85%. After 250 iterations, the prediction accuracy of SVM model is 65%, while the prediction accuracy of this model algorithm is 91%. The results show that the prediction accuracy of this model is higher than other models.

Fig 7 shows that the prediction precision of different types of bank marketing ability prediction models increases with the increase of model iterations. When the number of model iterations is 100, the prediction precision of SVM model is 38%, while that of KNN model is 45%, and that of ensemble learning model is 61%. After 250 iterations, the prediction precision of SVM model, KNN model and ensemble learning model is 41%, 49% and 69% respectively. By comparing the prediction precision curves of different models, the performance of this model is better than other models.

In Fig 8, when the number of model iterations is increased to 100, the change data of the predicted recall rate of several different types of bank marketing ability prediction models are constantly rising. When the model is iterated for 100 times, the prediction recall rate of the bank marketing ability evaluation model based on SVM is 44%, while the prediction recall rate of the bank marketing ability evaluation model based on KNN is 54%, and the prediction recall rate of the K-means model is 64%. The prediction recall rate of the model based on ensemble learning proposed in this paper is 73%. When the number of iterations of the model exceeds 100, the recall value tends to be basically stable. When the number of model iterations is 250, the prediction recall rate of the model based on ensemble learning is 74%. Therefore, the comprehensive performance of this model is better than other models.

In Fig 9, when the number of model iterations increases to 100 times, the predicted F1 value data of the whole system shows an overall growth trend. When the model is iterated for 100 times, the predicted F1 value of the banking marketing system based on SVM is 41%, while the predicted F1 value of the model based on KNN is 49%, and the predicted F1 value of the ensemble learning model proposed by the research can reach 75%. However, when the number of iterations of each model is 100 to 250 times, the F1 value of each model algorithm tends to be basically stable. When the iteration number of the model is 250, the predicted F1 value of the banking marketing system based on SVM is 50%, while the predicted F1 value based on the ensemble learning model proposed in this paper is 74%.

Through the analysis of the above results from accuracy, precision, recall and F1value, it is found that the prediction accuracy of the model algorithm in this paper is up to 91%. This may be due to the in-depth understanding of the characteristics of banking marketing in this model, and the integration of XGBoost algorithm and random forest algorithm in ensemble learning can capture the nonlinear relationship or highly complex patterns in the data, so the model algorithm in this paper can improve the generalization ability of the model and obtain the optimal prediction accuracy. However, random forest and SVM are different in data distribution, parameter adjustment and category imbalance, so they show different accuracy.

### 4.2 System data transmission performance of different types of models

**Fig 10:**
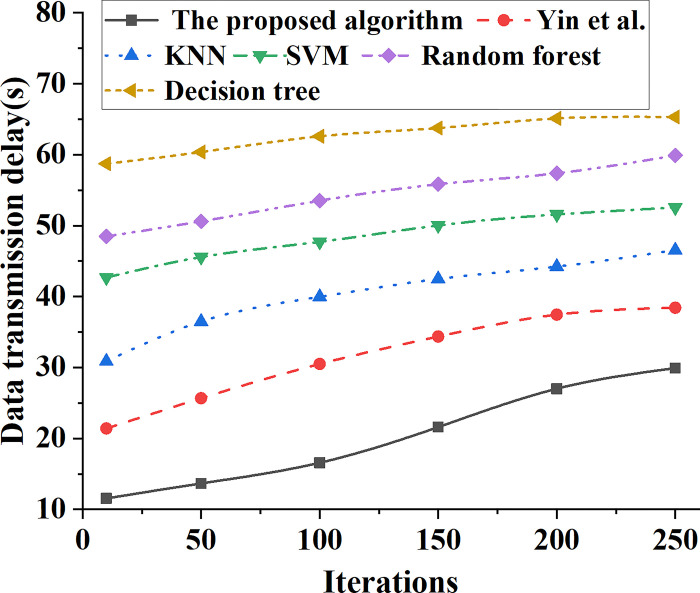
Data transmission delay curve of different types of bank marketing ability prediction models.

**Fig 11:**
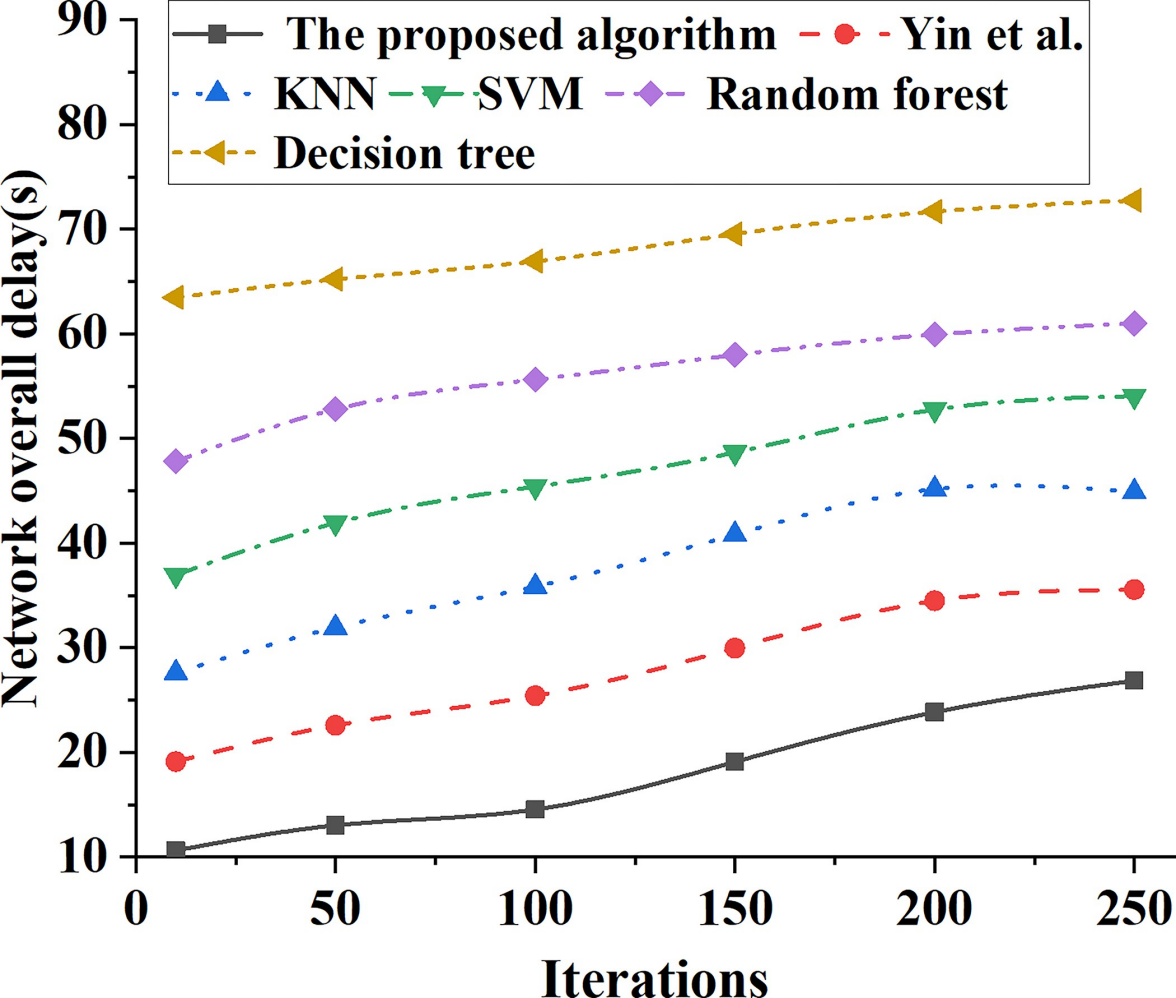
Numerical change curve of network overall delay of different types of bank marketing ability prediction models.

The data transmission performance of different types of models is analyzed. With the change of model iterations, the data transmission delay curves of different types of bank marketing ability prediction models are shown in Fig 10. In addition, the numerical change curves of the overall network delay of different types of bank marketing ability prediction models are shown in Fig 11.

In Fig 10, in different types of bank marketing ability prediction models, the data transmission delay of different models fluctuates with the increase of iteration times. When the number of model iterations is 50, the data transmission delay of the prediction model based on decision tree is 60s, that of the prediction model based on SVM is 46s, and that of the proposed model is only 14s. After 250 iterations of the model, the data transmission delay of the prediction model based on SVM is 52s, while the data transmission delay of the prediction system based on ensemble learning model proposed in this paper is only 30s.

In Fig 11, with the increasing number of network iterations, the overall network data transmission delay of the model is increasing. When the iteration number of the model is 50, the system transmission delay of the banking marketing forecasting system based on SVM is 42s, while the network transmission delay of the banking marketing system based on KNN algorithm is 32s. At this time, the network data transmission delay of K-means banking marketing system is 23s, while the network data delay of the proposed banking marketing system is only 13s. After 250 model iterations, the system transmission delay of the banking marketing forecasting system based on SVM is 54s, while the whole network transmission delay of the forecasting system based on the ensemble learning model proposed in this paper is only 27s. Comparing the comprehensive performance of different models, the overall data transmission performance of the bank marketing system based on ensemble learning model is better than other models.

Further, the model algorithm in this paper is compared with other model algorithms in terms of sales growth rate and customer satisfaction, and the results are shown in Table 1.

**Table 1:** Results table of sales growth rate and customer satisfaction growth rate of each model algorithm.

	Sales growth rate (%)	Customer satisfaction growth rate (%)
The proposed algorithm	25.67	20.52
Yin et al.(2022)	21.36	18.74
KNN	18.92	15.37
SVM	14.55	12.18
Random forest	16.78	14.29
Decision tree	13.99	11.85

In Table 1, the model algorithm in this paper shows the highest results in sales growth rate and customer satisfaction growth rate, which are 25.67% and 20.52% respectively. In contrast, the results of other algorithms are lower than the model algorithm in this paper, among which Yin et al. (2022) takes the second place in terms of sales growth rate and customer satisfaction growth rate, which are 21.36% and 18.74% respectively. Therefore, the relatively good effect of this model in the prediction of bank marketing ability can help banks better understand and improve their marketing ability.

### 4.3 Discussion

The results of this paper show that the proposed algorithm has achieved remarkable success in forecasting and improving the marketing ability of banks. Meanwhile, through this paper, its impact on bank marketing strategy is as follows:

First, the effectiveness of marketing ability prediction is improved. The algorithm in this paper shows excellent ability in forecasting the growth rate of sales and customer satisfaction. High sales growth rate and customer satisfaction growth rate show that the algorithm can effectively predict which marketing strategies and activities may be successful and have a positive impact on customers, which is consistent with Sugiato et al. (2023) [36]. This helps banks allocate resources and energy wisely to achieve better business performance.

Second, the accuracy of marketing decisions is improved. By using this algorithm, banks can predict the effect of marketing activities more accurately to make better decisions. Accurate marketing forecast helps to avoid the waste of resources, optimize advertising and promotion activities, and better meet customer needs. This is expected to improve the market competitiveness of banks.

Third, the efficiency of marketing decisions is improved. Through accurate forecasting, banks can make decisions more quickly, reduce the cost of trial and error, and adjust market strategies more flexibly. This helps banks adapt to market changes more quickly and seize business opportunities.

## 5. Conclusion

New marketing strategies, particularly those leveraging internet technologies, have significantly transformed the banking marketing landscape with the emergence of e-commerce technologies. This paper aims to delve into the data interaction process of the ensemble learning model through the utilization of machine learning and ensemble learning techniques. Based on the analysis of the traditional marketing model of banks, this paper builds an automatic learning model to analyze and predict customer data. The bank marketing system may carry out targeted marketing for customers, further sustain the relationship between customers and banks, and increase the likelihood of successful product marketing by contrasting the prediction models of random forest and SVM. The findings highlight the potential to establish a differentiated banking product marketing system using a forecasting model based on ensemble learning, further enhancing the market segmentation strategy of e-commerce banks. However, this paper also has some shortcomings. If the time span of data collection may be short, the data in a longer time range can be considered in future research to enhance the prediction performance of the model. In the future, in order to further improve the forecasting effect and marketing ability of the model, it is necessary to modify the model parameters through grid search in the future research. Moreover, the accuracy and practicability of the model can be further improved by further exploring the field of bank marketing ability prediction.

## Supporting information

S1 Data (ZIP)
