# Supplementary material for: Enhancing bank marketing strategies with ensemble learning: Empirical analysis
Source: PLoS One. 2024 Jan 11;19(1):e0294759. doi: 10.1371/journal.pone.0294759 (PMC10783788; doi:10.1371/journal.pone.0294759)
Supplement: S1 Data — (ZIP) [file pone.0294759.s001.zip › Data packet/code description.docx]

**Code configuration and preparation:**

Import the necessary libraries: At the beginning of the code, the required Python libraries are first imported. Make sure that you have installed these libraries, and you can use PIPIInstall to install them, such as PIPIInstall Numpy Pandas SCI Kit-Learn XG Boost. Load data: use pd.read_csv to load the bank marketing data set. Ensure that the dataset file is loaded with data according to the file path.

Data preprocessing: In practical application, you need to perform data preprocessing, including feature engineering, data cleaning, missing value processing and so on. This part of the code is not specifically shown in the example, because it depends on your data and problems.

**Partition data set:**

Divide features and target variables: Use data.drop and data["target"] to separate features and target variables. Ensure that the target variable in the dataset exists as a "target" column.

Divide training set and test set: use the train_test_split function to divide the data set into training set and test set. The data is divided into 80% training set and 20% test set. The random_state parameter is used to ensure that the randomness of the partition can be reproduced.

**The first stage: random forest feature selection and preliminary prediction**

Create a random forest model: use RandomForestClassifier to create a random forest classifier, and set the n_estimators parameter to 100, which means that 100 decision trees are used to build a random forest. Training random forest model: use rf_classifier.fit method to train random forest model on training set. Feature selection: The random forest model can obtain the importance score of each feature through the feature_importances_ attribute, and the importance score is used for feature selection here.

Filter Features: Based on the importance score of features, the sample code uses a threshold (0.01) to filter important features. Only features with an importance score greater than the threshold are retained.

**The second stage: model training with XGBoost.**

Create XGBoost model: use xgb.XGBClassifier to create XGBoost classifier, and also set the n_estimators parameter to 100.

Train XGBoost model: train XGBoost model on the filtered features by using xgb_classifier.fit method. Model evaluation: the XGBoost model is evaluated by using the test set, and the accuracy of the model is calculated here.

**Final forecast:**

Final forecast: The final code part is used to forecast and output the final bank marketing ability.

**The specific super parameters are as follows:**

The value of max_ depth is 4.

Gamma value is 0.7.

Min_ child- weight is 5.

Reg_ alpha is 0.01.

Reg_lambda is 100.

Num_leaves is 15.
